# Frecuencia de enteroparásitos en primates Cebidae y Callitrichidae del Zoológico de Cali, Colombia: implicaciones zoonóticas

**DOI:** 10.7705/biomedica.5403

**Published:** 2021-05-31

**Authors:** Jorge Iván Zapata-Valencia, Sebastián Ortega-Valencia, Yisther Katherine Silva-Cuero, Lina Sofía Castillo-Castillo, Laura Sofía Ortega-Ruiz, Adriana Cardona-Ortiz, Juliana Peña-Stadlin

**Affiliations:** 1 Escuela de Bacteriología y Laboratorio Clínico, Facultad de Salud, Universidad del Valle, Cali, Colombia Universidad del Valle Escuela de Bacteriología y Laboratorio Clínico Facultad de Salud Universidad del Valle Cali Colombia; 2 Área de Bienestar Animal, Fundación Zoológico de Cali, Cali, Colombia Fundación Zoológico de Cali Cali Colombia

**Keywords:** animales de zoológico, primates, parásitos, zoonosis, Cebidae, Callitrichidae, Animals, zoo, primates, parasites, zoonoses, Cebidae, Callitrichidae

## Abstract

**Introducción.:**

Los enteroparásitos pueden generar problemas en animales bajo cuidado humano en zoológicos y centros de acogida. Los animales silvestres presentan bajas cargas parasitarias, pero estas pueden ser mayores y llevar a manifestaciones clínicas cuando se trata de animales resguardados en recintos, lo que aumenta los gastos en tratamientos y cuidados médicos. Por otro lado, algunos enteroparásitos pueden causar infecciones zoonóticas en los cuidadores, los visitantes y otros animales del zoológico, así como afectar los programas de recuperación de especies amenazadas de extinción.

**Objetivos.:**

Determinar la presencia y prevalencia de enteroparásitos con potencial de transmisión zoonótica en primates de las familias Cebidae y Callitrichidae del Zoológico de Cali, entre septiembre y noviembre de 2017.

**Materiales y métodos.:**

Se hizo un estudio transversal prospectivo, para lo cual se recolectaron muestras seriadas de 50 individuos pertenecientes a siete especies de dos familias de primates y se analizaron mediante examen coprológico, flotación y coloración Kinyoun, entre septiembre y noviembre de 2017.

**Resultados.:**

Según su prevalencia, los géneros de parásitos hallados en las siete especies de primates evaluadas, fueron *Blastocystis* spp., *Trichomonas* spp., *Giardia* spp., *Entamoeba* spp., *Strongyloides* spp., *Cyclospora* sp. y *Trichuris* sp.

**Conclusiones.:**

Por lo menos, seis de los géneros de parásitos identificados tienen implicaciones zoonóticas, lo cual hace necesario establecer las posibles vías de infección de los primates del Zoológico de Cali e implementar protocolos de manejo que reduzcan el riesgo de transmisión a los humanos y a otros animales de la colección. Además, se presenta la información relevante sobre el potencial zoonótico de los enteroparásitos hallados.

## Abstract (en)

**Introduction:**

Enteroparasites can cause problems in animals kept under human care in zoos and shelters. Wild animals have low parasitic loads but when sheltered in closed places they can be higher and lead to clinical manifestations, which increases the cost of medical treatments and care. On the other hand, some enteroparasites can represent a potential risk of zoonotic infection for their animal keepers, visitors, and other zoo animals. In addition, they could affect recovery programs for endangered species.

**Objectives:**

To establish the presence and prevalence of potentially zoonotic enteroparasites in primates of the Cebidae and Callitrichidae families at the *Zoológico de Cali* from September to November, 2017.

**Materials and methods:**

We conducted a prospective cross-sectional study. Serial samples from 50 individuals belonging to seven species and two primate families were analyzed by ova and parasite test, flotation, and Kinyoun stain between September and November, 2017.

**Results:**

In order of frequency, the parasite genera found in the seven primate species evaluated were *Blastocystis spp.,Trichomonas* spp., *Giardia* spp., *Entamoeba* spp., *Strongyloides* spp., *Cyclospora* sp., and *Trichuris* sp.

**Conclusions:**

At least six of the parasite genera found have potential zoonotic implications. It is necessary to establish what are the infection sources at the *Zoológico de Cali* and implement management protocols to reduce the risk of transmission to both humans and other animals in the collection. Additionally, we offer relevant information on the zoonotic potential of each of the enteroparasites found.

Los primates no humanos se encuentran amenazados por acciones como la reducción drástica de sus hábitats, la fragmentación de los bosques, la caza ilegal y el tráfico de fauna silvestre, lo que lleva a que cerca del 60 % esté en peligro de extinción ^1^^-^^5^. Las especies neotropicales representan el 34 % de las especies de primates del planeta ^4^.

Colombia ocupa el quinto lugar de diversidad de primates no humanos del Nuevo Mundo ^2^, pero varias especies endémicas están amenazadas ^1^. Para mitigar este impacto, el Zoológico de Cali participa en programas de educación ambiental, conservación y recuperación de especies amenazadas, y recibe animales mediante el intercambio con otros zoológicos o el rescate de manos de los traficantes de fauna silvestre por parte de las autoridades ambientales.

Es frecuente encontrar una alta prevalencia de parásitos y comensales intestinales que causan morbilidad y mortalidad en los primates bajo cuidado humano ^6^^,^^7^. Los parásitos intestinales más comúnmente reportados son organismos unicelulares, hongos, nematodos, platelmintos y acantocéfalos, y es común que los animales tengan dos o más especies simultáneamente. Las prevalencias de algunos de ellos llegan a más del 35 % y varían en el tiempo debido a factores bióticos y abióticos ^8^^-^^12^.

El parasitismo intestinal en los primates no humanos puede causar síntomas como diarrea, cólicos, vómitos, prolapso rectal, fiebre, daño mecánico, malabsorción de nutrientes, pérdida de electrolitos y, en casos graves, obstrucción intestinal, lo que provoca la pérdida de su condición corporal y cambios de comportamiento que indican que su enfermedad cursa de manera grave y que, incluso, puede llevarlos a la muerte ^9^ y afectar los programas de recuperación de especies en peligro.

Algunas especies de *Cryptosporidium* presentes en animales bajo cuidado humano ^11^ pueden ocasionar infecciones intestinales y extraintestinales en niños e individuos inmunocomprometidos ^12^^,^^13^. *Entamoeba histolytica* en primates y humanos y *E. nuttalli* en primates se consideran extremadamente patógenas y causan infecciones extraintestinales que pueden ser mortales ^14^^-^^16^. *Strongyloides* spp. se ha informado como una causa importante de morbilidad en animales bajo cuidado humano, lo que incrementa los gastos de atención médica ^17^, en tanto que *Blastocystis* spp. y *Giardia* spp. son frecuentes en animales silvestres y en aquellos bajo cuidado humano, incluidos los primates no humanos, así como en el hombre, lo cual revela su potencial zoonótico ^18^^-^^23^.

En este estudio, se determinaron la presencia y la prevalencia de enteroparásitos con potencial de transmisión zoonótica en primates de las familias Cebidae *(Cebus capucinus, Sapajus apella* y *Saimiri sciureus)* y Callitrichidae *(Cebuella pygmaea, Saguinus leucopus, S. oedipus* y *S. fuscicollis)* del Zoológico de Cali, entre septiembre y noviembre de 2017.

## Materiales y métodos

El estudio, de tipo transversal prospectivo, se llevó a cabo en el Zoológico de Cali, entre septiembre y noviembre del 2017. El zoológico está situado en el bosque municipal, a orillas del río Cali, en la ciudad de Santiago de Cali (Valle del Cauca, Colombia), a una altura de 1.000 m.s.n.m. y con una precipitación anual promedio de 1.483 mm. Alberga alrededor de 2.500 animales, el 21 % de ellos mamíferos. Las dos familias de primates tenían 50 individuos distribuidos en 19 recintos con uno a nueve individuos cada uno.

En total, fueron 32 hembras (64 %), 13 machos (26 %) y cinco individuos sin sexo determinado (10 %).

Los cuidadores recolectaron en tres ocasiones las muestras de materia fecal de cada recinto, con intervalos inferiores a dos semanas entre recolecciones, empleando una técnica aséptica, guantes y recipientes limpios en cada ocasión. En los recintos ocupados por grupos no era posible realizar el muestreo de forma individual, por lo que se tomaron hasta cuatro muestras frescas simultáneas que se consideraron representativas del total de los animales de esa unidad de muestreo ^17^. Las muestras se recogían de aquellas deposiciones que estuvieran sobre el piso de concreto del recinto, evitando las que estaban sobre la tierra o el césped.

Los recipientes se rotularon adecuadamente y se llevaron al laboratorio del zoológico antes de una hora al cabo de la recolección. Se procesaron para la detección de enteroparásitos por examen coprológico directo (solución salina y lugol), concentración por flotación con sulfato de cinc y tinción ácido-alcohol resistente (Kinyoun) y se observaron con objetivos de 10X, 40X y 100X, según la técnica ^24^. La identificación se hizo con base en las características morfológicas de los microorganismos.

Los resultados se ingresaron en una base de datos en Excel, y se determinaron frecuencias y medidas de tendencia central. Con el paquete IBM SPSS™, statistics (versión 23), se estudió si había asociación o independencia entre las variables "familia de primate" e "infección" en cada una de las parasitosis encontradas, empleando la prueba exacta de Fisher con un nivel de significación estadística establecido (α) de 0,05.

### Aspectos éticos

El estudio fue aprobado por el Comité Institucional de Revisión Ética con Animales en Experimentación de la Universidad del Valle mediante el Acta No. 001-017 del 31 de mayo de 2016, y por la Fundación Zoológico de Cali.

## Resultados

Se tomaron muestras seriadas completas en 16 recintos, todos positivos para parasitismo intestinal en el momento del estudio y se descartaron tres recintos en los cuales se hospedaban diez animales, ya que en ellos no fue posible tomar la totalidad de las muestras del seriado. Así, el grupo evaluado quedó conformado por 40 individuos y el 100 % presentó una entidad parasitaria, por lo menos.

En cuanto a la riqueza parasitaria por especie de primate, *Saguinus leucopus* (Callitrichidae) fue la que presentó la mayor variedad de parásitos (dos nematodos *Trichuris* sp. y un adulto sin identificar, y seis protozoos de *Giardia* spp., *Cyclospora* spp., *Blastocystis* spp., *Trichomonas* spp., *Entamoeba* spp., y uno sin identificar). Le siguieron *Saimiri sciureus* (Cebidae), con cuatro parásitos *(Blastocystis* spp., *Trichomonas* spp., *Entamoeba* spp. y *Strongyloides spp.), Sag. oedipus,* con tres *(Giardia* spp., *Blastocystis* spp. y *Trichomonas* spp.) y *Cebuella pygmaea* (Callitrichidae), con tres *(Blastocystis* spp., *Trichomonas* spp. y *Entamoeba* spp.). Por último, los cébidos *Cebus capucinus (Blastocystis* spp.) y *Sapajus apella (Giardia* spp.), y el calitrícido *Sag. fuscicollis (Blastocystis* spp.), presentaron un tipo de enteroparásito.

En el análisis etario, la mayor prevalencia de infección se presentó en los animales mayores de 11 años, seguidos de los menores de un año y los de 13 meses a 5 años (cuadro 1). El microorganismo de mayor frecuencia según el número de individuos parasitados fue *Blastocystis* spp., seguido por *Trichomonas* spp., *Giardia* spp., *Entamoeba* spp. *(E. histolytica/E. dispar/E. moskovskii/E.nuttalli), Strongyloides* spp., *Cyclospora* sp. y *Trichuris* sp. Por otro lado, en el análisis por recintos, los parásitos o comensales intestinales más prevalentes fueron *Blastocystis* spp., *Trichomonas* spp., *Entamoeba* spp. (E. *histolytica/E. dispar/E. moskovskii/E. nuttalli)* y *Giardia* spp. Los de menor frecuencia fueron *Strongyloides* spp., *Trichuris* sp. y *Cyclospora* sp. (cuadro 2).

**Cuadro 1:** Distribución etaria de primates Cebidae y Calitrichidae analizados y total de individuos parasitados

Edad	n	Total de individuos	%
0-12 meses	4	5	80
13 meses a 5 anos	8	10	80
6-10 años	9	13	69,2
≥11 años	8	9	88,8
Indeterminada	2	3	66,7

**Cuadro 2:** Prevalencia de parásitos en primates Cebidae y Calitrichidae por número de individuos infectados y su distribución por recintos en valores absolutos y porcentajes

Parásito	Parásitos o comensales por número de individuos (N=40) n (%)	Parásitos o comensales por recintos (N=16) n (%)
*Blastocystis* spp.	30 (75)	14 (84,2)
*Trichomonas* spp.	11 (27,5)	5 (31,3)
*Giardia* spp.	11 (27,5)	3 (18,8)
*Entamoeba* spp. (*Entamoeba histolytica/E. dispar/E. moskovskii/E. nuttalli*)	10 (25)	4 (25)
*Strongyloides* spp.	6 (15)	1 (6,3)
*Cyclospora* sp.	2 (5)	1 (6,3)
*Trichuris* sp.	1 (2,5)	1 (6,3)

En las 22 hembras, los 13 machos y los 5 individuos de sexo indeterminado, se identificaron agentes parasitarios o comensales. Al analizar el número de recintos y la cantidad de especies parasitarias en cada uno de ellos, así como el número de especies parasitarias en las dos familias de primates, se pudo apreciar que el monoparasitismo era la condición más frecuente tanto en los recintos como por familia, seguido por el poliparasitismo de dos y tres especies, y con menor frecuencia, el poliparasitismo de cuatro especies. En los cuadros 3 y 4 se pueden apreciar las asociaciones entre las diferentes especies de parásitos en los individuos con infecciones múltiples por familia y por recinto.

**Cuadro 3:** Prevalencia de parasitismo en primates Cebidae y Calitrichidae por sexo y número de individuos, en valores absolutos y porcentajes

Análisis por sexo				
Sexo	Animales por sexo n	Tipo de parasitismo	n	%
Hembras	22	Monoparasitismo	14	63,6
		Poliparasitismo, 2 especies	2	9,1
		Poliparasitismo, 3 especies	2	9,1
		Poliparasitismo, 4 especies	4	18,2
Machos	13	Monoparasitismo	5	38,5
		Poliparasitismo, 2 especies	6	46,2
		Poliparasitismo, 3 especies	1	7,7
		Poliparasitismo, 4 especies	1	7.7
Indeterminado	5	Monoparasitismo	5	100

**Cuadro 4:** Asociación de especies en individuos con poliparasitismo y su distribución por recintos

Tipo de infección	Total de recintos afectados (N=16) n	Número de recintos por tipo de parasitismo n	Parásitos	%
Monoparasitismo	7	6	*Blastocystis* spp.	42,8
		1	*Giardia* spp.	
Poliparasitismo, 2 especies	4	2	*Blastocystis* spp.	25
			*Trichomonas* spp.	
		2	*Blastocystis* spp.	
			*Entamoeba* spp.	
Poliparasitismo, 3 especies	4	1	*Trichuris* sp.	25
			*Giardia s*pp.	
			*Trichomonas* spp.	
		1	*Blastocystis* spp.	
			*Cyclospora* spp.	
			Protozoo sin identificar	
		1	*Blastocystis* spp.	
			*Entamoeba s*pp.	
			Nematodo adulto sin identificar	
		1	*Blastocystis* spp.	
			*Trichomonas* spp.	
			*Giardia* spp.	
Poliparasitismo, 4 especies	1	1	*Blastocystis* spp.	6,2
			*Entamoeba* spp.	
			*Strongyloides* spp.	
			*Trichomonas* spp.	

**Figura 1:**
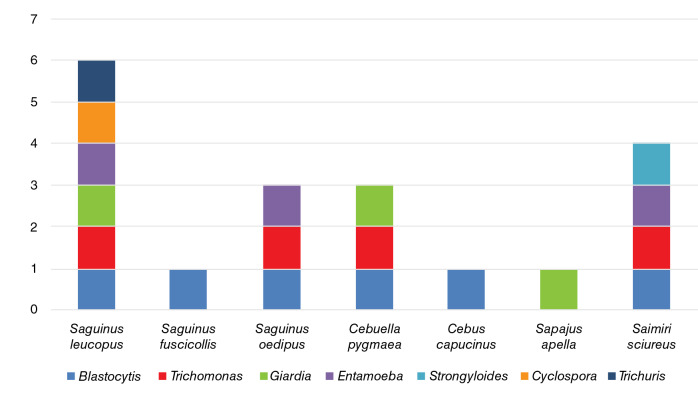
Diversidad de enteroparásitos (riqueza de especies parásitas) en los primates de las familias Cebidae y Callitrichidae en el Zoológico de Cali *(Blastocystis* spp., *Trichomonas* spp., *Giardia* spp., *Entamoeba* spp., *Strongyloides* spp., *Cyclospora* sp. y *Trichuris* sp.)

Al comparar los parásitos según la cantidad de especies huéspedes que pueden infectar, *Blastocystis* spp. se encontró en un mayor número de especies de primates, con seis distintas especies de huéspedes en las dos familias de primates no humanos del Nuevo Mundo. *Trichomonas* spp. se encontró en cuatro especies de primates, en tanto que *Giardia* spp. y *Entamoeba* spp. se encontraron en tres huéspedes cada una y, *Trichuris* sp., *Cyclospora* sp. y *Strongyloides* spp., solo en una especie cada una (cuadro 5). Al comparar la cantidad de especies parasitarias encontradas en cada especie de primate, *Sag. leucopus* (n=8) presentó seis distintos parásitos *(Trichuris* sp., *Giardia* spp., *Cyclospora* sp., *Blastocystis* spp., *Trichomonas* spp. y *Entamoeba* spp.), siendo los de mayor diversidad de microorganismos, mientras que la menor variedad de parásitos ocurrió en *Sag. fuscicollis* (n=1), *C. capucinus* (n=6) y *Sap. apella* (n=9), con una especie de parásito cada uno *(Blastocystis* spp. en las dos primeras especies y *Giardia* spp. en la otra) (figura 1 y cuadro 5).

**Cuadro 5:** Número de individuos positivos Vs. número total de individuos por parásito para las diferentes especies de las familias Cebidae y Callitrichidae del Zoológico de Cali

Especie	***Blastocystis* spp.**	***Trichomonas* spp.**	***Giardia* spp.**	***Entamoeba* spp.**	***Strongyloides* spp.**	***Cyclospora* sp*.***	***Trichuris* sp*.***
*Saguinus leucopus*	7(8)	3(8)	1 (8)	2(8)	0(8)	2(8)	1 (8)
*Saguinus fuscicollis*	1 (1)	0(1)	0(1)	0(1)	0(1)	0(1)	0(1)
*Saguinus oedipus*	4(4)	1 (4)	0(4)	2(4)	0(4)	0(4)	0(4)
*Cebidae*							
Cebuella pygmaea*	6(6)	1 (6)	1 (6)	0(6)	0(6)	0(6)	0(6)
*Cebus capucinus*	6(6)	0(6)	0(6)	0(6)	0(6)	0(6)	0(6)
*Sapajus apella*	0(9)	0(9)	9(9)	0(9)	0(9)	0(9)	0(9)
*Saimirí sciureus*	6(6)	6(6)	0(6)	6(6)	6(6)	0(6)	0(6)
Total	30 (40)	11 (40)	11 (40)	10 (40)	6 (40)	2 (40)	1 (40)

El número en paréntesis corresponde al total de Individuos de la especie.

* No se incluyeron los primates sin el seriado completo.

En los recintos en los que se obtuvieron las tres muestras seriadas, mediante el primer examen se diagnosticaron infecciones parasitarias en siete (43,8 %), en tanto que los otros nueve recintos fueron negativos. De estos últimos, ocho fueron positivos en la segunda muestra. El acumulado de positivos para las dos primeras muestras fue del 93,8 %. En el recinto que albergaba al grupo de individuos de *Sap. apella,* las dos primeras muestras fueron negativas y, en la tercera, el resultado fue positivo para *Giardia* spp. (cuadro 6). De los tres recintos en los que solo se recolectó una muestra, dos fueron positivos para *Blastocystis* spp. y el otro fue negativo.

**Cuadro 6:** Muestras de materia fecal positivas en las distintas tomas del examen seriado (datos por recinto)

Muestra en la cual se detectaron los parásitos	Número de recintos positivos (N=16)^*^ n	%	% acumulado
1	7	43,8	43,8
2	8	50,0	93,8
3	1	6,2	100,0

* De los 19 recintos hubo tres en donde solo se obtuvo una muestra.

En la prueba exacta de Fisher, tomando como muestra el número de recintos, (n=16), se encontró que las variables "familia de primate" y cada una de las parasitosis encontradas no tenían asociación estadísticamente significativa, es decir, eran independientes.

## Discusión

En el presente estudio se determinó la presencia y la prevalência de enteroparásitos con potencial de transmisión zoonótica en primates de las familias Cebidae y Callitrichidae del Zoológico de Cali. En aquellos espacios con más de un individuo, se consideró que el riesgo de exposición era similar para todos y que los resultados eran aplicables a todos los primates del recinto ^17^. La frecuencia de infección parasitaria en las especies de primates evaluadas fue del 100 %, similar a lo reportado en otros zoológicos en Perú ^7^ y Colombia ^25^, ambas mayores del 95 %, pero mayor que la reportada en otros zoológicos de Bélgica ^17^, China ^18^, Medellín ^26^, España ^27^^)^ y Costa Rica ^28^, así como en estudios con animales silvestres libres en Costa Rica ^29^, Perú ^30^ y Colombia ^31^, todos con frecuencias entre el 25 y el 75 %.

Cabe destacar el estudio de Fajardo, *et al.,* realizado también en el Zoológico de Cali, en el cual se encontró una prevalencia de parasitismo mucho menor de *Entamoeba* spp. y uncinarias en *C. capucinus* (6,35 % cada uno), en tanto que no se encontró parasitismo en los ejemplares de *Sag. leucopus*^32^. Es importante mencionar que, en algunos de los trabajos referenciados, se utilizó una única toma de muestra ^1^^,^^7^^,^^25^^,^^30^, lo que permite pensar que la sensibilidad en el diagnóstico de las infecciones con bajas cargas parasitarias fue menor, mientras que, en este trabajo, se evaluaron muestras seriales para todos los recintos, con el fin de mejorar la sensibilidad del examen directo y de la coloración.

Debe señalarse que los animales que el Zoológico de Cali recibe por intercambio o decomiso entran en cuarentena en un área independiente, durante la cual se les hacen diversas evaluaciones clínicas periódicas para establecer su estado de salud, incluidos exámenes sanguíneos y estudios seriados de materia fecal. Pasado este periodo, se aplica el protocolo para su introducción como individuos fundadores y no se los introduce en grupos ya establecidos para, así, garantizar la inocuidad de los nuevos individuos que se integran a la colección del Zoológico.

Como los parásitos detectados en los primates evaluados en este estudio son transmitidos principalmente por agua y alimentos, con excepción de *Strongyloides* spp., que penetra por la piel, es importante señalar que el Zoológico cuenta con su propia planta de potabilización de agua con cloro para el suministro a los diversos recintos; sin embargo, hay que tener en cuenta que varias especies de enteroparásitos presentan estructuras resistentes a la cloración del agua, por lo que para algunos animales especialmente propensos se ha establecido un protocolo de potabilización por ósmosis inversa que reduce el riesgo de infección por *Toxoplasma gondii*^33^. Con respecto a las frutas y verduras que se emplean para la alimentación de los primates, estas se manejan con protocolos similares a los establecidos para la manipulación de los alimentos para consumo humano y son suministrados en bandejas plásticas que se lavan después de cada uso.

Por otra parte, el protocolo de manejo de enteroparásitos en el Zoológico de Cali implica la evaluación de cada recinto tres veces al año mediante examen coprológico directo y concentración. Cuando se encuentran individuos positivos, se establece el tratamiento y se evalúa la necesidad de tratar los recintos adjuntos; si hay individuos sintomáticos, por ejemplo, por diarrea, se hace el diagnóstico, se da tratamiento grupal y, solo en condiciones especiales, dadas las implicaciones sociales, se aísla al individuo para su manejo en la clínica bajo observación continua.

Los recintos de los primates en el Zoológico de Cali cuentan con una zona de manejo con superficies lavables, así como zonas con hierba para simular condiciones naturales. Debido a la dificultad de su lavado, estas zonas pueden actuar como reservorio para las formas infectivas de los parásitos gastrointestinales, lo que facilita su permanencia y viabilidad en el tiempo, y la reinfección de los individuos, y explicaría las altas tasas de prevalencia halladas en este y otros estudios ^3^^,^^17^^,^^21^. Por otra parte, es difícil controlar el ingreso de animales, como pequeñas aves, roedores e insectos (cucarachas y moscas) a los recintos; estos animales pueden volverse parte de la dieta de los primates bajo cuidado humano y los convierte en un factor de riesgo de infección parasitaria ^34^^,^^35^.

La prevalencia general de helmintos en las dos familias de primates fue baja comparada con otros estudios, lo que se explicaría por las bajas cargas parasitarias de los individuos y porque el examen directo no es la mejor técnica para el diagnóstico de las infecciones por *Strongyloides* spp. Asimismo, podría tener relación con la propensión de los huéspedes a la infección, o con su comportamiento, y con los protocolos de manejo implementados en el zoológico ^31^^,^^36^. En estudios de cébidos en vida silvestre en Costa Rica ^37^^,^^38^ y Colombia ^1^^,^^31^, los parásitos de mayor frecuencia fueron helmintos, incluidos acantocéfalos, y con menor frecuencia, los protozoarios, hecho que algunos autores explican como resultado de la dieta de algunos de estos primates, la cual puede incluir invertebrados que servirían de huéspedes intermediarios para algunos de estos helmintos ^31^^,^^37^^-^^39^.

En cuanto a la frecuencia de monoparasitismo (43,8 %) hallada en los cébidos y calitrícidos del Zoológico de Cali, esta fue similar a lo reportado en un estudio realizado en Costa Rica (60,8 %) ^28^, pero difiere de lo informado en estudios llevados a cabo en otros zoológicos ^17^^,^^40^. La elevada frecuencia de monoparasitismo podría indicar también que, aunque las fuentes de infección son pocas, la simplicidad de los ciclos de vida de los parásitos detectados, monoxénicos, y el hecho de que sus estadios quísticos *(Entamoeba* spp., *Blastocystis* spp., *Giardia* spp.) son infectivos una vez son excretados por el huésped, facilitan que un animal se reinfecte después del tratamiento, ya que en algunos de los recintos es difícil limpiar totalmente la materia fecal. Ahora bien, si se compara el poliparasitismo en estos primates (25 % como máximo) con el hallado en otros zoológicos, la frecuencia fue menor ^41^. Esto es importante porque las infecciones por múltiples especies parasitarias suelen causar cuadros clínicos más complicados en los individuos infectados y, por ende, aumentar los costos en tratamientos y cuidados.

El estrés causado por la permanencia en espacios restringidos y el contacto con seres humanos, se ha propuesto como una de las causas de la gran frecuencia de infecciones parasitarias intestinales en primates bajo cuidado humano. Se asocia con la reducción de la resistencia a las infecciones y el aumento de la cronicidad de estas parasitosis ^42^. Por ello, en el Zoológico de Cali se han intervenido los recintos siguiendo los protocolos establecidos en manuales de bienestar específicos para cada familia de primates ^43^^-^^45^, de manera que su diseño facilite un manejo y una interacción adecuados con el cuidador, y un mayor bienestar de los animales. Ello permitió que el Zoológico fuera certificado por la *Association of Zoos and Aquariums* (AZA) y la Asociación Latinoamericana de Parques Zoológicos y Acuarios (ALPZA). Además, se asigna un único cuidador para la alimentación y el aseo de los recintos para reducir el estrés en los animales a su cargo y el ingreso a las áreas de manejo se hace mediante motivación positiva, lo que se consigue con el diseño y la ambientación, y con estrategias de condicionamiento positivo como los premios.

Por último, cabe resaltar la utilidad del examen seriado de materia fecal que, en este caso, permitió detectar más del 90 % de los casos de infección en la segunda muestra evaluada, algo similar a lo reportado en otros estudios ^28^. En algunos de los primates no humanos del Nuevo Mundo, es frecuente que la muestra sea escasa o insuficiente para utilizar las técnicas rutinarias debido a la cantidad de materia fecal que expulsan, por lo cual la repetición de la toma de muestras múltiples en los recintos aumenta considerablemente la probabilidad de observar estadios parasitarios en el examen coprológico.

### Sobre el potencial zoonótico de los parásitos hallados en primates Cebidae y Callitrichidae

Es importante resaltar el potencial zoonótico de algunos de los enteroparásitos encontrados en los primates del Zoológico de Cali, ya que podrían ser fuente de infección para sus cuidadores y para otros animales de la colección. En el caso de *Blastocystis* spp. y *Trichomonas* spp., no se ha definido claramente su papel en la enfermedad en animales y, a pesar de su elevada frecuencia en los primates evaluados, la mayoría estaban asintomáticos. Por su parte, *Giardia* spp. un protozoo frecuente en los animales del Zoológico de Cali, y es conocido su papel en la enfermedad animal y humana. En cuanto a *Strongyloides* spp., se presentó en pocos individuos de las dos familias de primates *Cebidae y Callitrichidae;* en el Zoológico se han presentado casos recurrentes de estrongiloidiasis crónica en lémures, que podrían ser un foco de infección para humanos.

Entre los enteroparásitos unicelulares, *Blastocystis* spp. es uno de los más frecuentemente hallados en muestras fecales de personas sintomáticas y asintomáticas ^23^^,^^46^. Sin embargo, todavía se debate su papel patogénico, aunque se han descrito casos de enfermedad diarreica y otros trastornos gastrointestinales y extraintestinales en personas en las que no se encuentra otro agente patógeno, incluidos el síndrome de colon irritable y la enfermedad inflamatoria del colon ^46^^-^^49^. A pesar de la elevada prevalencia en los animales del Zoológico de Cali y en otros, estos usualmente no presentan sintomatología, por lo que se sugiere que no es agente patógeno para los animales ^48^. Se han descrito 17 subtipos de *Blastocystis* spp. en diferentes huéspedes vertebrados, incluidos varios primates, y en el hombre ^23^^,^^49^^-^^51^ se han aislado 10 subtipos (ST1-ST9 y ST12) ^52^. *Blastocystis* spp. fue el parásito de mayor prevalencia en las dos familias de primates en este estudio (84,2 %), similar a lo reportado en otras investigaciones ^23^^,^^49^^,^^50^^,^^53^. También, se ha reportado en cébidos y calitrícidos en zoológicos y reservas naturales de Perú, Chile y Colombia ^26^^,^^30^^,^^54^^-^^57^. En los estudios en Colombia con técnicas moleculares, se halló una baja prevalencia de *Blastocystis* spp. en primates silvestres del Magdalena Medio ^31^, lo que indicaría que el estar bajo cuidado humano los expone a este organismo, en tanto que, en condiciones silvestres, tiene menor relevancia este parásito.

Se han llevado cabo algunos estudios de caracterización genética de *Blastocystis* spp., principalmente a partir de aislamientos provenientes de primates del Viejo Mundo ^23^^,^^30^^,^^53^^,^^58^^,^^59^. En algunos zoológicos europeos, se determinó que los ejemplares de la familia Callitrichidae *(Saguinus labiatus* y *Callithrix jacchus)* presentaban los subtipos 1 y 3, dos de los más frecuentes en humanos, mientras que los del Viejo Mundo presentaron los subtipos 1, 2, 3, 5 y 8, entre otros, y con prevalencias mayores que las observadas en los del Nuevo Mundo ^58^^-^^61^.

Aunque humanos y primates comparten diferentes subtipos, hay variación en la frecuencia de los patrones de alelos entre ellos en los humanos, predomina el alelo 4 del subtipo 1 y, en los primates, se encuentran seis alelos distintos, más frecuentes que el 1 y el 2. Al igual que en otras investigaciones ^22^^,^^58^^,^^61^, en un par de muestras de cuidadores de los zoológicos evaluados en uno de los estudios, se encontraron alelos que coincidieron con los observados en primates (alelos 2 y 6), lo que demuestra que puede haber transmisión zoonótica entre los animales y sus cuidadores. Esta situación es similar a la observada con el subtipo 3 y sus alelos, los cuales difieren entre humanos y primates, aunque en los primates ninguno de los alelos presenta una frecuencia dominante, lo que refleja que no hay una especificidad por estos huéspedes ^60^.

En otros estudios se ha demostrado la posible transmisión zoonótica de los subtipos 1, 2 y 8; este último es bastante raro en humanos, excepto en cuidadores de zoológicos ^22^^,^^60^^,^^62^. Aunque se comparten los subtipos, hay diferencias en los alelos entre los aislamientos de *Blastocystis* spp. de primates bajo cuidado humano y los de humanos. Sin embargo, no se ha determinado cuáles son los alelos circulantes en poblaciones silvestres y si también se presentan en humanos.

La tricomoniasis intestinal es frecuente en los vertebrados terrestres del Zoológico de Cali (anfibios, reptiles, aves y mamíferos) y se diagnostica rutinariamente en los controles periódicos de todos los animales de la colección en los que participan dos de los autores de este trabajo; sin embargo, parece ser de poca relevancia en los primates, aunque se han detectado individuos parasitados en vida silvestre y bajo cuidado humano. En Costa Rica se informó su presencia hasta en el 11 % de un grupo de cébidos silvestres *(C. capucinus)*^29^ y, en el presente estudio, la frecuencia fue mayor, de 31 %.

Aunque en el momento del estudio ninguno de los animales infectados presentó alteraciones gastrointestinales, en el 2015 hubo en el Zoológico de Cali un caso fatal de tricomoniasis intestinal en un lémur *(Varecia variegata)* con diarrea crónica y linfangiectasia. Este individuo presentó una infección persistente con elevada carga parasitaria y fue tratado por uno de los autores sin lograrse la curación completa por resistencia al tratamiento con metronidazol. Por otra parte, en un primate del Nuevo Mundo de la familia Pithecidae *(Callicebus moloch),* se reportó un caso de tricomoniasis invasiva que se manifestó con depresión, diarrea y deshidratación. La histolopatología reveló la presencia de abundantes trofozoítos en diversas áreas del colon y en los ganglios linfáticos mesentéricos; el parásito se identificó mediante microscopía electrónica, sin evidencia de otro agente patógeno ^63^.

Actualmente, se aceptan nueve especies de *Trichomonas* diferenciadas principalmente por su morfología, muchas de ellas descritas en primates del Viejo y el Nuevo Mundo en un único reporte ^64^; entre ellas, está *Tritrichomonas mobilensis,* descrita en *Saimiri boliviensis boliviensis,* un primate del Nuevo Mundo ^65^. En uno de los estudios en que se han utilizado técnicas moleculares para determinar cuántas especies de *Trichomonas* intestinales infectan distintos huéspedes, se estudiaron 10 especies de primates del Viejo Mundo y un calitrícido (C. *jacchus);* 25 aislamientos de dichos primates *(Hylobates syndactylus, Pantroglodytes, Lemur catta, Macaca silenus, M. nigra, Mandrillus sphinx* y *Semnopithecus entellus)* se agrupaban en varios grupos o linajes del género *Tetratrichomonas,* y los autores propusieron separarlos, por lo menos, en ocho especies ^64^. En este mismo grupo, se alineó un flagelado aislado de un paciente humano con empiema pleural ^66^, en tanto que un aislamiento de *L. catta* se asoció con *T. gallinarum,* especie que se ha aislado de la cavidad oral, los bronquios y el esputo de seres humanos ^67^. Un aislamiento de *Otolemur garnettii* se asoció con *Hypotrichomonas acosta,* un flagelado encontrado en serpientes, y otro de *L. catta* se relacionó estrechamente con *Trichomitus batrachorum*, un género de anfibios y reptiles emparentado con *Hypotrichomonas* en Hypotrichomonadea (Parabasalia).

Por último, la secuencia de tres aislamientos de *Colobus angolensis, V. variegata* y *C. jacchus* fue muy similar a la reportada para *Pentatrichomonas hominis* en ganado y humanos ^64^. Estos hallazgos revelan-que la diversidad de parabasálidos en primates es mayor de la estimada y plantea la necesidad de estandarizar las técnicas moleculares para la correcta identificación de los trichomonadidos intestinales presentes en primates bajo cuidado humano, ya que pueden tener potencial zoonótico, como se ha demostrado con *Tetratrichomonas* y *P. hominis;* esta última puede causar enfermedad bajo ciertas condiciones en animales silvestres bajo cuidado humano, en humanos y en animales domésticos (Núñez J, Zerpa R, Lucas CM, Lugo-Román LA, Gregory MJ, Maves RC, *et al. Pentatrichomonas hominis* is associated with diarrheal episodes in captive-bred owl monkeys *(Aotus nancymaae).* 61st Annual Meeting. November 11-15, 2012. Atlanta, GA, USA: American Society of Tropical Medicine and Hygiene; 2012) ^68^^-^^73^. Es necesario caracterizar genéticamente las cepas de *Trichomonas* spp. circulantes en el Zoológico de Cali para conocer las especies, y poder establecer si hay riesgo zoonótico y las medidas que minimicen la transmisión a otros animales de la colección y a los cuidadores.

La prevalencia de *Entamoeba* spp. (25 %) indica que es un parásito importante en los primates de las familias Cebidae y Callitrichidae del Zoológico de Cali. Otros estudios en zoológicos reportan prevalencias de 28,9, 81,1 y 100 % ^18^^,^^74^^,^^75^.

En primates del Nuevo y el Viejo Mundo, se han descrito varias especies de este género, como *Entamoeba histolytica, E. nuttali, E. chattoni (E. polecki* de subtipo 2), *E. polecki, E. hartmanni, E. coli, E. moshkovskii y E. dispar,* de las cuales solo las dos primeras se han reportado como causantes de sintomatología intestinal y extraintestinal en algunos animales infectados ^16^^,^^19^^,^^41^^,^^74^^-^^77^. Entre las especies de primates consideradas, solo se ha reportado *E. chattoni (E. polecki* de subtipo 2) en uno del Nuevo Mundo *(Alouatta palliata,* Atelidae) en Costa Rica ^78^, pero su descripción se basó en la comparación morfológica y morfométrica, y no en técnicas moleculares que lo confirmaran.

Con el empleo de zimodemos y PCR, se han detectado infecciones por *E. nuttalli* y *E. chattoni (E. polecki* de subtipo 2), mayoritariamente asintomáticas, en cuidadores de animales en zoológicos europeos ^15^^,^^41^^,^^79^. De ahí la importancia de la determinación molecular de las especies de *Entamoeba* observadas en los animales analizados en este estudio ya que, de tratarse de *E. nuttali* o *E. histolytica,* pueden llegar a causar enfermedad clínica, ocasionalmente fatal. Asimismo, es necesario establecer los factores de riesgo asociados a las infecciones en estos animales, para reducir la transmisión tanto entre los primates como a otras especies del zoológico ^79^ o a sus cuidadores ^15^.

En humanos, *G. duodenalis* es uno de los principales protozoos causantes de diarrea, especialmente en niños ^80^. Actualmente, se han identificado ocho grupos (A-H) de esta especie, morfológicamente indistinguibles, pero genéticamente distintos, en diversos mamíferos ^81^, y algunos autores proponen separarlos como especies ^82^. Se ha visto que los grupos A y B de *G. duodenalis* tienen múltiples huéspedes y potencial zoonótico ^83^. Este protozoo se considera un agente patógeno importante en primates, en los que ocasiona diarrea y retraso en el crecimiento de animales jóvenes ^9^^,^^74^^,^^84^^,^^85^.

En distintos países, los primates del Nuevo y el Viejo Mundo en condición silvestre o bajo cuidado humano en zoológicos, presentan estos parásitos, con prevalencias hasta del 94 % ^31^^,^^37^^,^^74^^,^^86^^-^^89^. No obstante, son pocos los estudios moleculares de aislamientos provenientes de primates, destacándose los realizados en Italia y España en primates del Viejo Mundo, en los que se identificaron genotipos de *Giardia* spp. pertenecientes al grupo B, subgrupo BIV, también hallado en humanos ^74^^,^^88^^-^^90^, lo que sugiere que pueden tener potencial zoonótico.

Sin embargo, se ha demostrado que la transmisión de *Giardia* spp. puede ser antroponótica o zoonótica bidireccional, de manera que los primates pueden ser reservorios para el humano, pero los seres humanos también inciden en la contaminación ambiental con quistes de *Giardia* spp. ^81^^,^^90^. Hasta el momento no se han caracterizado genéticamente los aislamientos de *Giardia* spp. provenientes de primates en el Zoológico de Cali, por lo que se desconoce el verdadero riesgo zoonótico para los cuidadores y otros animales de la colección.

*Trichuris trichiura* es un nematodo que causa enfermedad en humanos y primates ^91^^,^^92^; se asume que solo esta especie infecta tanto a unos como a otros ^93^ y se reporta con frecuencia en primates no humanos del Nuevo y del Viejo Mundo en estado silvestre ^29^^,^^30^^,^^93^^-^^95^ o bajo cuidado humano ^9^^,^^27^^,^^87^^,^^96^^,^^97^, con prevalencias hasta del 100 % en algunas especies del Viejo Mundo, y entre 1,94 y 11,64 % en algunas del Nuevo Mundo bajo cuidado humano, incluidos cébidos y calitrícidos ^8^.

En S. *fuscicollis* (Callitrhricidae) silvestres de Colombia y Brasil, se han encontrado prevalencias del 50 y el 14 %, respectivamente, en tanto que, en *Cebus versicolor* (Cebidae) silvestres de Perú y Brasil, se ha informado una de 64,3 % ^29^^,^^30^^,^^96^. En primates bajo cuidado humano en Colombia, *T. trichiura* se ha reportado en *S. leucopus*^54^, y en Chile, en *C. albifrons*^57^. En el Zoológico de Cali se detecta ocasionalmente en diversos mamíferos en coinfección con *Capillaria* sp., un helminto de aves.

En algunos estudios de caracterización de aislamientos de *Trichuris* sp. en primates no humanos del Viejo Mundo mediante diferentes marcadores moleculares, se detectó la presencia de *T. trichiura*^98^^,^^99^. En un reporte de Uganda en primates silvestres de áreas de contacto frecuente con humanos, se encontraron varios linajes de *Trichuris* sp. con elevada variabilidad genética y evidencia de que, al menos, uno de ellos era compartido por humanos y primates no humanos, lo que podría significar que tiene potencial zoonótico ^93^^,^^99^.

En un estudio posterior, al analizar el genoma mitocondrial de aislamientos provenientes de humanos, cerdos y primates del Viejo Mundo bajo cuidado humano, se apreció una gran variabilidad y que los genotipos de primates y humanos formaban cinco y dos clados separados, respectivamente, distantes de los de cerdo. Se ha propuesto, entonces, que hay un complejo de especies crípticas que infectan a humanos y primates, y que algunas de ellas tienen gran especificidad de huéspedes, pero otras pueden infectar a unos y otros ^93^^,^^99^. Aún se requieren estudios que permitan caracterizar genéticamente los parásitos encontrados en los primates no humanos del Nuevo Mundo para establecer si son especies distintas a *T. trichiura* y si tienen potencial zoonótico.

Otro parásito que se reporta con frecuencia en varios grupos de vertebrados, entre ellos los primates y el humano, es el nemátodo *Strongyloides* spp. ^10^^,^^100^^-^^104^. Hay tres especies descritas que infectan a los humanos; una es S. *stercoralis,* que se considera cosmopolita, y las otras dos presentan una distribución geográfica más restringida. S. *fuelleborni fuelleborni* causa zoonosis en primates y humanos de África y Asia, y S. *fuelleborni kellyi* infecta humanos de Papúa Nueva Guinea ^104^^-^^106^.

*Strongyloides stercoralis* puede cursar como una infección asintomática o causar cuadros clínicos complicados de hiperinfección o diseminación que pueden llegar a ser mortales, especialmente en animales jóvenes y en humanos inmunosuprimidos o con infección por el virus linfotrópico de células T humanas de tipo 1 (HTLV -1) ^105^^-^^107^. En el hemisferio occidental, solo se han informado dos casos humanos fatales de infección por *S. fuelleborni,* en Perú, en dos mujeres de 5 y 16 años, ambas positivas para infección con HTLV-1, que presentaron un cuadro diarreico, infección diseminada y sepsis bacteriana. El diagnóstico se basó en la identificación morfológica de los huevos en cadena con membrana envolvente y hembras adultas de vida libre con constricción posvulvar característica ^106^^-^^108^. Sin embargo, en Suramérica no se han descrito huéspedes naturales para *S. fuelleborni.*

Coincidente con los resultados del presente estudio, en primates no humanos del Viejo y del Nuevo Mundo bajo cuidado humano o silvestres, incluidos cébidos y calitrícidos de Colombia, Ecuador, Perú, Chile y Costa Rica, se han presentado infecciones por *Strongyloides* spp. Con frecuencias que fluctúan entre 1,94 y 66 % ^8^^,^^17^^,^^23^^,^^25^^,^^26^^,^^29^^,^^30^^,^^37^^,^^42^^,^^78^^,^^109^^-^^111^. En cuanto a la infección en los del Nuevo Mundo, en algunos trabajos se ha reportado la presencia de *S. cebus* o *Strongyloides* sp. ^42^, pero sin que se hubieran empleado técnicas moleculares para establecer la especie involucrada en las infecciones detectadas. En Costa Rica se ha reportado en *C. capucinus, C. albifrons* y *C. apella,* silvestres y bajo cuidado humano, con prevalencias del 10 al 36 % ^29^^,^^37^^,^^38^. En zoológicos de Chile y Perú, se ha informado en cébidos y calitrícidos *(Saguinus mystax, S. fuscicollis, S. sciureus, C. apella y C. albifrons)*^30^^,^^39^^,^^42^^,^^57^ y, en Colombia, en grupos de *C. versicolor* y S. *leucopus* de vida silvestre, con prevalencias entre 18,8 y 62,5 % ^1^^,^^31^. *Callithrixpenicillata* se ha empleado como un modelo biológico para evaluar la infección por *S. stercoralis* en individuos con inmunosupresión, lo que ha permitido observar que en estos primates la estrongiloidiasis se presenta con cuadros clínicos que van desde los no complicados hasta la condición grave, similar a lo que sucede en humanos ^112^, lo que demuestra que esta especie de primates es sensible a la infección por este nemátodo.

Debe tenerse en cuenta que la infección natural por *S. stercoralis* en primates silvestres del Viejo Mundo puede estar ligada al hecho de compartir su hábitat con humanos u otros animales que pueden portar el parásito, y además, que la infección puede llegar a causar una enfermedad fatal ^113^. Algunos estudios en estos primates, en los cuales se han empleado la caracterización y diferenciación molecular de *S. stercoralis* y *S. f. fuellborni,* han revelado la variabilidad genética de este parásito y su carácter zoonótico ^102^^,^^104^^,^^114^. Una de las secuencias analizadas de S. *f. kellyi* es idéntica a una sequencia de S. *cebus.* La información obtenida con este gen y la de las secuencias SSU, permite separar dos clados, uno conformado por *S. ratti, S. suis, S. venezuelensis, S. vituli* y S. *papillosus,* y el otro basado en las secuencias de *S. f. fuelleborni,* que agrupa esta especie con secuencias idénticas de aislamientos de *S. stercoralis* a partir de humanos y perros, y de *Strongyloides* sp. de serpientes ^102^^,^^112^^,^^115^.

Con base en estos hallazgos, se propone que S. *f. kellyi* puede ser una especie *(Strongyloides kellyi)* independiente de S. *fuelleborni*^104^^,^^112^. A pesar de que se ha demostrado que los humanos pueden estar infectados con diversos genotipos de *S. stercoralis,* y de que hay transmisión zoonótica de esta y otras especies, es necesario continuar estudiando a los primates no humanos del Nuevo Mundo para aclarar si las especies de *Strongyloides* presentes en ellos también se comportan de manera similar a las encontradas en los del Viejo Mundo.

Comparada con la reportada en algunos estudios, la prevalencia de *Strongyloides* spp. en el Zoológico de Cali fue baja, lo que puede estar asociado con los esquemas de tratamiento programados para los animales y su efectividad. Asimismo, podría estar relacionada con el subdiagnóstico de aquellos animales con cargas parasitarias bajas que no son detectados con métodos de poca sensibilidad como el examen coprológico directo, lo que hace necesario la implementación de técnicas que mejoren el diagnóstico, como el cultivo en agar o el embudo de Baermann o, idealmente, pruebas moleculares o dispositivos como el Flotac que no se emplearon en este estudio ^104^^,^^105^^,^^111^^,^^113^^,^^114^.

En cuanto a la coccidia intestinal *Cyclospora cayetanensis,* se asocia con cuadros diarreicos en humanos, especialmente en países tropicales, donde afecta principalmente a niños y pacientes inmunosuprimidos, en tanto que. en los países desarrollados. se ha descrito principalmente en brotes por alimentos y como agente de diarrea del viajero ^116^. En primates no humanos, *Cyclospora* spp. se informa principalmente en los del Viejo Mundo, tanto silvestres como bajo cuidado humano en zoológicos, con prevalencias entre el 2,5 y el 68 % ^27^^,^^117^^-^^120^.

*Cyclospora cayetanensis*^121^ fue la primera especie descrita en primates humanos; posteriormente. se describieron tres nuevas especies (C. *cercopithecide*, *C. colobi* y *C. papionis)* en *Cercopithecus aethiops, Colobus guereza* y *Papio anubis* en estado silvestre, respectivamente ^122^. El integrante más reciente de este grupo de parásitos intestinales de primates es *C. macacae,* aislada de *Macaca mulatta*^119^. En Colombia y en México, se han reportado unos pocos casos de *Cyclospora* spp. en especies de primates silvestres del Nuevo Mundo de la familia Atelidae *(Alouatta seniculus, A. pigra* y *Ateles geoffroyi)*^120^^,^^123^. En ambos estudios la identificación se hizo mediante coloración ácido-alcohol resistente, pero no se hizo la caracterización molecular de los aislamientos. Hasta el momento, no se han reportado casos de transmisión de la infección por especies de *Cyclospora* de primates a humanos y, dado que este organismo está emparentado con *Eimeria,* es posible que tenga una gran especificidad de huéspedes, razón por la cual la infección cruzada sería muy rara ^40^^,^^122^.

Con base en la información disponible sobre el potencial zoonótico de los distintos parásitos observados en estos primates del Zoológico de Cali, se recomienda implementar técnicas de laboratorio que permitan identificar estas especies crípticas para contribuir al conocimiento de la fauna parasitaria de los primates neotropicales y dar cuenta de la posible transmisión zoonótica en el parque. Queda por esclarecer si las diferencias entre familias y las infecciones por *Blastocystis* spp., más prevalente en calitrícidos, y por *Giardia* spp. y *Strongyloides* spp., más frecuentes en cébidos, son hallazgos incidentales, o si hay alguna característica genética, inmunológica o de exposición que las explique; además, si varían según los periodos lluviosos o secos, o se mantienen a lo largo del año.

Se identificaron siete géneros parasitarios en primates Cebidae y Callitrichidae del Zoológico de Cali, con una mayor prevalencia de *Blastocystis* spp., *Giardia* spp. y *Trichomonas* spp., teniendo los dos primeros un potencial zoonótico considerable. El presente estudio reveló que los protocolos de evaluación periódica y el tratamiento oportuno seguidos en el zoológico parecen ser efectivos para reducir las infecciones por helmintos, ya que estas se presentaron en menor proporción. Dado que el monoparasitismo fue más frecuente, la presencia de las infecciones por parásitos unicelulares debe conducir a la evaluación de posibles fuentes de infección (alimentos, agua, calzado de los cuidadores y animales recién llegados al zoológico), para diseñar estrategias que minimicen su efecto.

Estos resultados demuestran que es necesario implementar técnicas de diagnóstico molecular para conocer de manera precisa las especies y los genotipos presentes en los animales de la colección, y dilucidar las fuentes de transmisión y los riesgos de zoonosis. Además, son de utilidad para los protocolos de vigilancia y tratamiento, encaminados a controlar la transmisión y el riesgo de contaminación entre los animales y sus cuidadores. Después de este estudio, se introdujeron cambios en el manejo de algunos recintos, como el reemplazo total y periódico del pasto y la tierra, así como la desinfección con vapor de las instalaciones y el aseo del calzado de los cuidadores. Aún está pendiente evaluar la efectividad de estas estrategias en la reducción de la contaminación y la infección por enteroparásitos.

Se demostró que el análisis seriado de muestras para el diagnóstico de parásitos intestinales en animales bajo cuidado humano, es útil para minimizar el subdiagnóstico que se puede presentar con la toma de una única muestra. Por último, teniendo en cuenta que estas especies de primates están en riesgo, el conocimiento de los parásitos que los afectan permite elaborar esquemas de tratamiento y control que garanticen animales aptos para reproducirse y programas para introducirlos en ecosistemas silvestres.
